# Editorial: Embodied Pedagogy and Movement (Discourses) in Physical Education

**DOI:** 10.3389/fspor.2022.873091

**Published:** 2022-05-17

**Authors:** Gunn Helene Engelsrud, Pirkko Markula

**Affiliations:** ^1^Institute of Sport, Food and Natural Science, Western Norway University of Applied Sciences, Sogndal, Norway; ^2^Faculty of Kinesiology, Sport, and Recreation, University of Alberta, Edmonton, AB, Canada

**Keywords:** physical education, higher education, teacher education, embodied pedagogy, bodily learning

We have invited a number of expert researchers in the field to contribute diverse “theoreticoempirical” positions. Their work illustrates the rich knowledge developed through qualitative methodology in practice-based projects as well as theoretical approaches on embodied learning in a variety of contexts. They creatively examine how individuals explore new movement abilities and create different physical activity experiences in juggling, unicycling, improvising dance, playing, engaging with standards of appearance, experiencing racism, being invited to find their favorite places, or creating meaning in the great outdoors—even if it is to get some fresh air on the balcony of an apartment building!

All authors both problematize and expand upon our knowledge about institutionalized pedagogical practices in PE, in higher education and in society. Their theoretical perspectives range from John Dewey, phenomenology of the lived and inter-affective bodies, embodied learning, critical whiteness theory and learning theory to Foucault's poststructuralism and Guattarian ideas about “genuine movement.”

Some authors problematize racism and white privilege in PE and PE teachers' focus on pre-defined sport instructions that should value diversity and intercultural competence. Others discuss the relative “absence” of the moving body in PE teaching that ironically should focus on movement education. When the body is involved, there tends to be a strong focus on bodily appearance. Several authors share an unwillingness to objectify movement as something bodies do or that is done to them in an instrumental fashion. Instead, the authors understand knowledge about embodied pedagogy as a way of being attuned to the pulse of life and choose to highlight the meaningful relational connections that can be formed in and through movement—and in doing so, they illuminate how bodily learning makes a person “interactive for life.” They investigate movement learning and learning *in* movement and replace the concept of life-long physical activity with life-long *interactivity*. In this context, movement learning development takes place when practitioners enhance their own way of participating in the activity. In concrete terms, learning to incorporate and activate one's skills is relevant to all embodied learning processes, including those that take place in sporting and non-sporting activities such as recreational mountain biking, expressive and creative dance, and improvisational practices.

The authors have contributed the wealth of critical and relational knowledge on embodied pedagogy and movement in PE. Their work has made us explore new research possibilities in movement pedagogy and we hope the readers will share our excitement. All we have left to say is READ ON!

## Author Contributions

GE wrote the draft editorial that PM edited and approved for publication.

## Conflict of Interest

The authors declare that the research was conducted in the absence of any commercial or financial relationships that could be construed as a potential conflict of interest.

## Publisher's Note

All claims expressed in this article are solely those of the authors and do not necessarily represent those of their affiliated organizations, or those of the publisher, the editors and the reviewers. Any product that may be evaluated in this article, or claim that may be made by its manufacturer, is not guaranteed or endorsed by the publisher.

